# Clinical trials in palliative care: a systematic review of their methodological characteristics and of the quality of their reporting

**DOI:** 10.1186/s12904-016-0181-9

**Published:** 2017-01-25

**Authors:** Raquel Bouça-Machado, Madalena Rosário, Joana Alarcão, Leonor Correia-Guedes, Daisy Abreu, Joaquim J. Ferreira

**Affiliations:** 1 Clinical Pharmacology Unit, Instituto de Medicina Molecular, Faculty of Medicine, University of Lisbon, Avenue Professor Egas Moniz, 1649-028 Lisbon, Portugal; 2 Center for Evidence-Based Medicine, Faculty of Medicine, University of Lisbon, Avenue Professor Egas Moniz, 1649-028 Lisbon, Portugal; 3 Laboratory of Clinical Pharmacology and Therapeutics, Faculty of Medicine, University of Lisbon, Avenue Professor Egas Moniz, 1649-028 Lisbon, Portugal

**Keywords:** Palliative care, Methodological quality, Risk of bias, Clinical trials

## Abstract

**Background:**

Over the past decades there has been a significant increase in the number of published clinical trials in palliative care. However, empirical evidence suggests that there are methodological problems in the design and conduct of studies, which raises questions about the validity and generalisability of the results and of the strength of the available evidence. We sought to evaluate the methodological characteristics and assess the quality of reporting of clinical trials in palliative care.

**Methods:**

We performed a systematic review of published clinical trials assessing therapeutic interventions in palliative care. Trials were identified using MEDLINE (from its inception to February 2015). We assessed methodological characteristics and describe the quality of reporting using the Cochrane Risk of Bias tool.

**Results:**

We retrieved 107 studies. The most common medical field studied was oncology, and 43.9% of trials evaluated pharmacological interventions. Symptom control and physical dimensions (e.g. intervention on pain, breathlessness, nausea) were the palliative care-specific issues most studied. We found under-reporting of key information in particular on random sequence generation, allocation concealment, and blinding.

**Conclusions:**

While the number of clinical trials in palliative care has increased over time, methodological quality remains suboptimal. This compromises the quality of studies. Therefore, a greater effort is needed to enable the appropriate performance of future studies and increase the robustness of evidence-based medicine in this important field.

**Electronic supplementary material:**

The online version of this article (doi:10.1186/s12904-016-0181-9) contains supplementary material, which is available to authorized users.

## Background

From the first time it was used, the concept of “palliative care” (PC) has suffered a series of transformations in how it is defined and consequently in the relevant area of operation and objectives [1, 2]. In 2002 the World Health Organization affirmed that PC improves the quality of life of patients and their families facing problems associated with life-threatening illness, through the prevention and relief of suffering by means of early identification and impeccable assessment and treatment of pain and other physical, psychosocial, and spiritual issues [1, 3].

Changes in demographic trends, including the ageing of populations and the increased life expectancy of individuals with life-limiting illnesses, have increased demand for high quality PC services. Today, the initiation of a treatment on the basis on what is believed to be effective is no longer considered good clinical practice [4]. A clinician in addition to his clinical expertise, must have access to the best available evidence, should carefully appraise its quality and assess its applicability to each individual patient [5, 6].

According to a MEDLINE search, the number of PC clinical trials (CT) published has quadrupled, from inception to 2005 [7, 8]. Whilst this may be beneficial, questions exist around the type and quality of the research being undertaken. Previous reviews have concluded that PC studies were largely descriptive, with a wide variation in sample size, in demographic and clinical aspects and with a lack of use of recognised standard measures and consideration of key outcomes [5, 6, 9–12]. Visser et al. [5] studied the reality of evidence-based practice in palliative care and highlighted additional problems like unpowered studies, recruitment difficulties and high attrition rates, inadequate duration of follow-up and difficulty in defining outcomes and avoiding performance bias [5, 9, 13].

In response to the high variability of clinical practice and the increasing costs and complexity of care, evidence is needed to define what are the most effective treatments. Good quality randomized controlled trials (RCTs) are the gold standard for evaluating the efficacy and effectiveness of health care interventions [14, 15]. Since previous publications showed a low number of randomized clinical trials (RCT) in the palliative care field, to achieve a more comprehensive view of therapeutic palliative care research, we designed a broad search strategy including all types of controlled clinical trials (CCT), of which RCT represent a subgroup [14, 15]. The goal of this systematic review was to evaluate the methodological characteristics of CCT in palliative care and to assess their quality of reporting.

## Methods

### Literature search

We performed a MEDLINE search through Ovid from inception (1946) to February 2015 using a pre-defined search strategy (Additional file 1) designed by the authors based on The Cochrane Collaboration’s highly sensitive search strategy to identify RCTs in the field of palliative care.

### Study selection

Inclusion criteria for studies were:

- prospective controlled clinical study;
- pharmacological and non-pharmacological interventions;
- studies evaluating palliative care interventions (according to each of the authors’ definition) conducted in patients and/or family members or caregivers, regardless the place of care;
- full-length article available.

We excluded:

- non-experimental studies (observational studies, systematic reviews, methodological studies, study protocols);
- experimental studies which did not evaluate palliative care interventions;
- experimental studies evaluating palliative care interventions not directly focused in patient-family dyad (cost-effectiveness analysis, evaluation of palliative care services/units, and interventions directed at health professionals).

Titles and abstracts of citations were independently pre-screened by two reviewers (RB, MR) according to review study selection criteria. The inclusion or exclusion criteria were applied and studies were selected for consideration on the basis of full text reports. Two reviewers independently assessed the full study reports; disagreements were resolved by consensus or by consultation with a third reviewer (JJF).

### Data extraction and quality assessment

Before study selection, a data extraction form with 43 items was developed, based on the checklist of guidelines for the design and evaluation of clinical trials (CONSORT, SPIRIT) [16–18]. Data extraction was done manually by two researchers (RBM, MR) without any extraction software. Five domains were analysed:

- general information (title of the CCT, name and country of the corresponding author, language of publication, year and journal of publication, journal impact factor, area and type of intervention, personal dimension and key points of practice of PC evaluated, ethical approval and informed consent);
- methods (eligible criteria, type of study design, method of randomisation, achievement of allocation concealment, type of blinding, and duration of follow-up);
- sample (intervention, total number of randomised patients and number of patients in each group, duration and timing of treatment, dropout rate, and sample size calculation);
- data analysis (type of analysis, statistical methods used, pre-defined outcomes, assessment tools, and group comparability);
- results.

Included articles were classified by clinical domain (e.g. oncology, neurology) and type of intervention. Four types of interventions were considered: pharmacological, non-pharmacological (all non-pharmacological interventions provided by health care professionals that are specifically mentioned as part of the interdisciplinary palliative care interventions [19]), non-pharmacological complementary therapies (all non-pharmacological interventions, such as musical and aromatherapy, that are not considered as part of the core palliative care interdisciplinary interventions [19]), and home-care based (all pharmacological and non-pharmacological interventions provided in patient’s home).

We identified PC milestones (focus on whole-person, patient and family empowerment, good communication, improvement of quality of life and teamwork) most relevant in the aims of each study. Based on them, we proceeded with two different types of classifications, one according to the main personal dimensions (physical, psychological, social or spiritual dimensions), and a second level in line with other factors of PC practice (communication, symptoms control, family support and team work) [20, 21].

The methodological quality of the included studies was assessed using the Cochrane Risk of Bias (RoB) tool [22]. This tool quantifies the association between certain design features and estimates of treatment effects. The RoB tool is a two-part instrument and includes the following areas: sequence generation, allocation concealment, blinding (of participants, investigators and outcome assessment), incomplete outcome data, selective outcome reporting and “other issues”. The first part refers to the description of what was reported in the trial, detailed enough for a judgement to be made based on this information. The second part appraises the risk of bias for each analysed area and classifies them in three categories: low, high or unclear risk of bias [15, 23].

Independently, two authors (RBM, MR) extracted information on individual items from all included studies and assessed the two parts in each study. Discrepancies were resolved through discussion or by consultation with a third reviewer (JJF).

### Statistical analysis

We summarised the publication characteristics using frequencies and percentages. Pooled odd ratios (OR) and the 95% confidence interval (CI) were calculated using a random effects model. This method offers summary estimates by combining the individual results published by independent researchers. It increases power and produces more precise summary estimates of the risk of dropout between interventions and control groups [24]. Differing dropout rates between treatment and control arms, with fewer patients being followed up in one arm than the other, increases the risk of attrition bias and the possibility of false-negative results [22, 25]. For this analysis we used Review Manager 5.3.0 software [22], Mantel-Haenzel method to account for the heterogeneity (clinical and methodological) among studies.

## Results

**Fig. 1:**
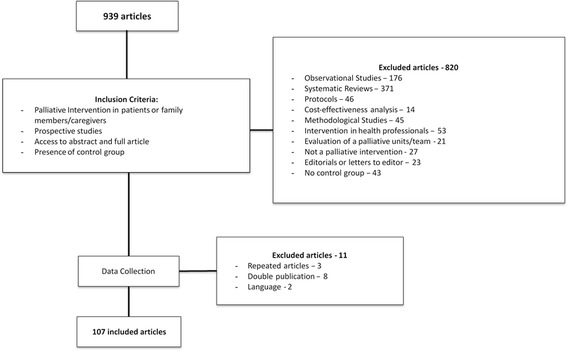
Flow diagram of study selection process

The electronic search identified 939 citations. After screening abstracts 120 articles were deemed potentially eligible. The application of inclusion criteria excluded 13 studies. The main reasons for exclusion were: repeated in the list of references (*n* = 3), duplicated publications (*n* = 8) and non-English language (*n* = 2) (Fig. 1).

### General features

**Fig. 2:**
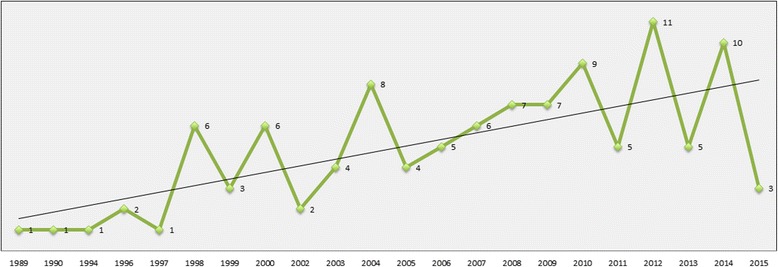
Number of clinical trials published over time

Of the 107 clinical trials included (Additional file 2), 12.2% (*n* = 13) were published between 1989 and 1999, 45.8% (*n* = 49) between 2000 and 2009, and 41.1% (*n* = 44) between 2010 and 2015 (Fig. 2). Studies were published in fifty-seven different journals, with the most reported being: Journal of Pain and Symptom Management (14.9%, *n* = 16, impact factor [IF]: 2.47), Palliative Medicine (13.1%, *n* = 14, IF: 2.85), Journal of Palliative Medicine (9.3%, *n* = 10, IF: 2.06) and Journal of Clinical Oncology (5.6%, *n* = 6, IF: 17.9). Most studies were conducted in the United States (USA) (26.2%, *n* = 28), the United Kingdom (UK) (21.5%, *n* = 23), Australia (11.2%, *n* = 12), and Canada (6.5%, *n* = 7). Fifteen percent (*n* = 16) of all the studies lacked mention of approval by an ethics committee.

### Types of design

Eighty-two point three percent (*n* = 88) of all the studies had a parallel design and 17.7% (*n* = 19) had a crossover design.

The most used comparator was non-intervention (control group participants did not receive any intervention for the duration of the study follow-up)/best supportive care (46.7%, *n* = 50) followed by placebo (27.1%, *n* = 29) and other interventions (25.2%, *n* = 27). The analysis of type of intervention and type of comparator demonstrated that non-intervention/best supportive care was essentially used in non-pharmacological interventions (80%, *n* = 40), while other interventions and placebo were more used in pharmacological interventions (88.9%, *n* = 24 and 62.1%, *n* = 18). Another intervention was chosen more often than placebo in pharmacological interventions.

Follow-up duration varied between studies. The most common periods were 1 month (14%, *n* = 15), 2 months and 2 weeks (9.3%, *n* = 10 each). The shortest follow-up was 30 min (at the end of an intervention) and 54 months was the longest period reported.

### Eligibility

Eligibility criteria varied significantly throughout studies. In the included studies all patients were at least 18 years old and no studies indicated the gender or ethnicity of participants. According to what has been previously reported, oncological disease is often an inclusion criterion. In three studies (2.8%), dementia was also an inclusion criterion, while it was an exclusion criterion in 29 studies (27.1%). The expected remaining lifespan of participants varied between “less than a week of life” and 24 months, with 6 months of life being the most commonly considered period. In 66.4% (*n* = 71) of articles this data was unknown.

### Clinical domains

**Fig. 3:**
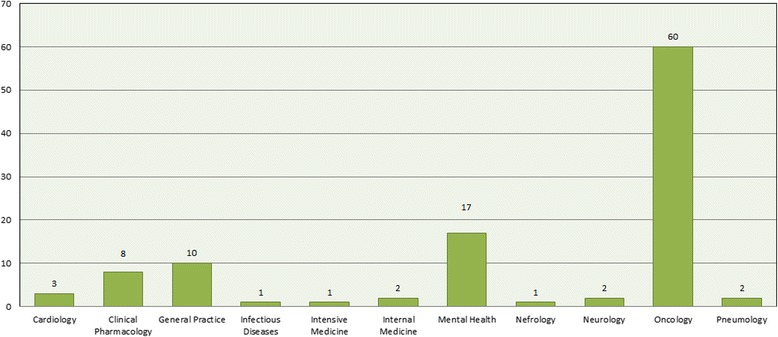
Distribution of included CTs across medical fields

The three clinical domains more present in the palliative care included studies were: oncology (56.1%, *n* = 60), mental health (15.9%, *n* = 17) and general practice (9.3%, *n* = 10) (Fig. 3).

### Types of interventions

**Fig. 4:**
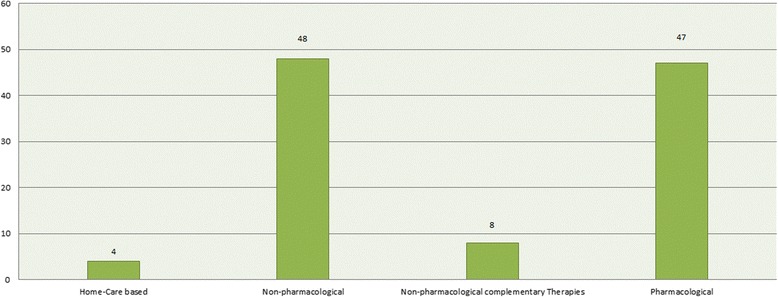
Distribution of included CTs based on types of intervention

Regarding the type of intervention, 44.9% (*n* = 48) of studies reviewed were non-pharmacological interventions, 43.9% (*n* = 47) pharmacological interventions, 7.5% (*n* = 8) non-pharmacological complementary therapy interventions, and 3.7% (*n* = 4) home-care based interventions (all pharmacological and non-pharmacological interventions provided in patient’s home. See Fig. 4).

### Palliative care classifications

With respect to the personal dimension studied, 63.6% (*n* = 68) analysed the physical dimension, 13.1% (*n* = 14) the psychological dimension, 14% (*n* = 15) the social dimension and 9.3% (*n* = 10) the spiritual dimension. By classifying the studies according to the other key points of palliative care practice we found that 70.1% (*n* = 75) of the studies were based on symptom control evaluation, teamwork and communication both represented 12.1% (*n* = 13) of studies, and 5.6% (*n* = 6) studies highlighted family support.

### Outcomes and assessment tools

As expected due to the broad scope of this review, there was a significant diversity of evaluated clinical outcomes. However, in 40.2% of the included trials (*n* = 43) no primary outcome was defined. When mentioned, the most cited primary outcomes were: pain intensity (20.3%, *n* = 13), improvement in quality of life (12.5%, *n* = 8), improvement in dyspnoea (9.4%, *n* = 6), and survival rate (7.8%, *n* = 5). The most common secondary outcomes were: improvement in quality of life (29.9%, *n* = 32), improvement in depression and anxiety (19.6%, *n* = 21), use of rescue doses or palliative care services (15.9%, *n* = 17), or presence of side effects (15%, *n* = 16). In the absence of a pre-specified main outcome, we considered all outcomes as secondary.

**Fig. 5:**
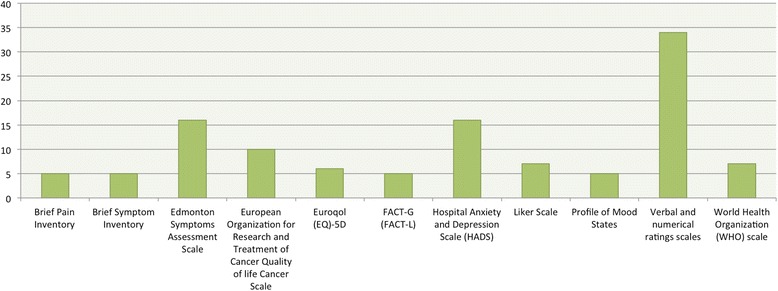
Distribution of the most used evaluation scales in included studies

For outcome assessment 137 different scales and questionnaires were used, with only eleven (8%) used in more than five studies. Twenty (14.6%) of the 137 are recommended by the National Palliative Care Research Center, 5 (3.7%) belong to the group of most used scales (Fig. 5).

### Statistic analysis

Four studies (3.7%) failed to describe statistical planning, only one (0.9%) used descriptive analysis. In the majority of studies the analysis per protocol was deduced from the presence of dropouts and the absence of intention-to-treat analysis reporting. Half the studies (50.5%, *n* = 54) used intention-to-treat analysis, 47.7% (*n* = 51) analysis per protocol and in two articles (1.9%) it was not possible to conclude which statistical analysis had been used. Sample size calculation was not indicated in 38.3% (*n* = 41) of studies, and 8.4% (*n* = 9) used a convenience sample. Of the 57 studies that presented a sample size calculation, in only 36.8% (*n* = 21) was the number of included patients above the estimated sample size.

### Dropouts

The mean sample size was 113.1 (SD 139.1) [range 9–820] participants, with a median of 64.5. The mean dropout frequency (*n* = 99 studies) was 22%, 40.2% of the studies had a dropout rate > 20% (cut-off used to assess risk of bias). The main causes of attrition were symptom burden and clinical deterioration. The clinical domains and types of intervention with a higher percentage of studies with a dropout rate > 20% were: oncology (*n* = 30, 28%), mental health (*n* = 11, 10.3%), pharmacological (*n* = 29, 27.1%), and non-pharmacological interventions (*n* = 23, 21.5%).

**Fig. 6:**
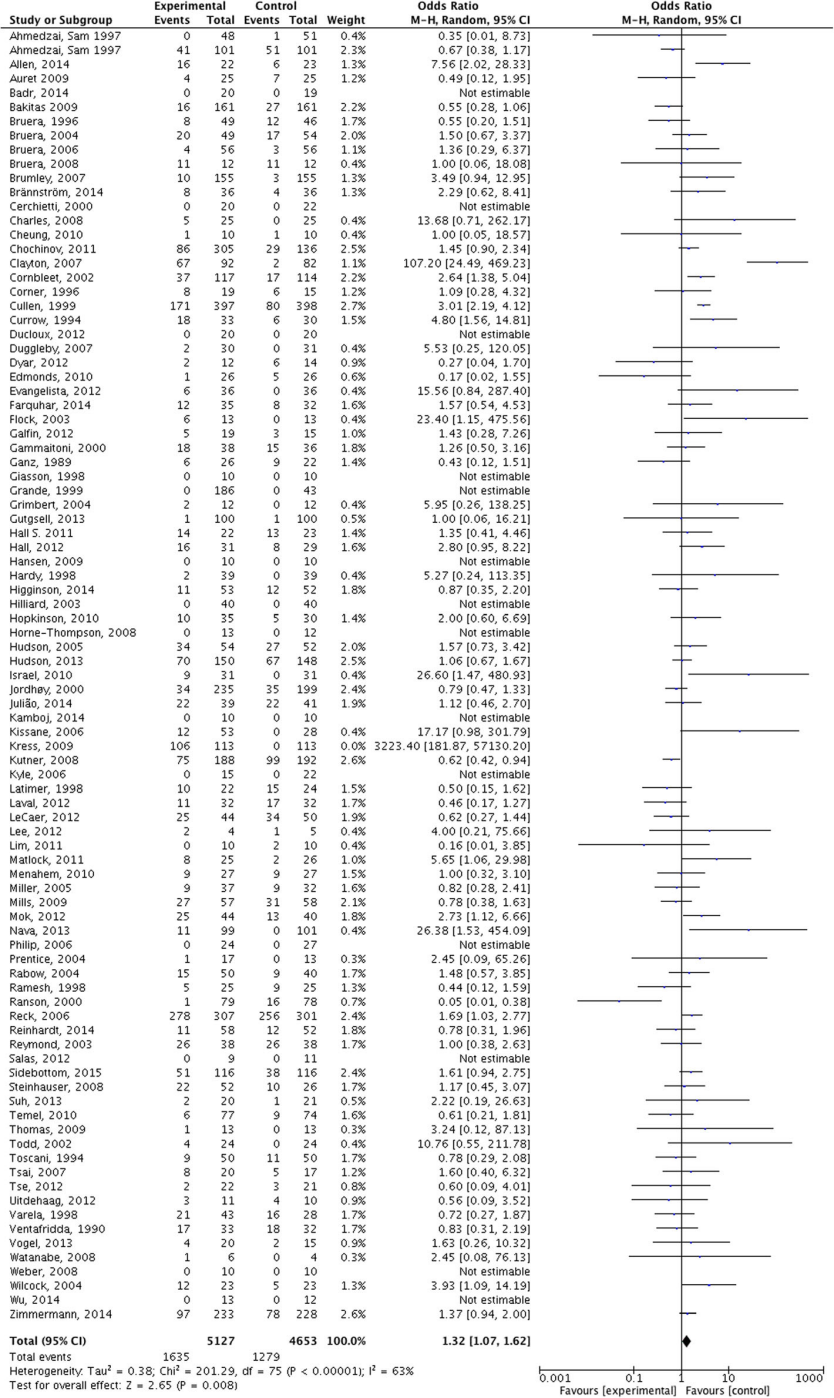
Forest plot comparing dropouts between intervention and control group

Pooled results from studies that reported one or more dropouts (*n* = 91) showed higher dropout rates among the active intervention groups (OR 1.32; 95% CI 1.07, 1.62). However, despite the use of a random effects model, the high level of heterogeneity limits the accuracy of the meta-analysis results (Fig. 6).

### Quality of reporting analysis

**Fig. 7:**
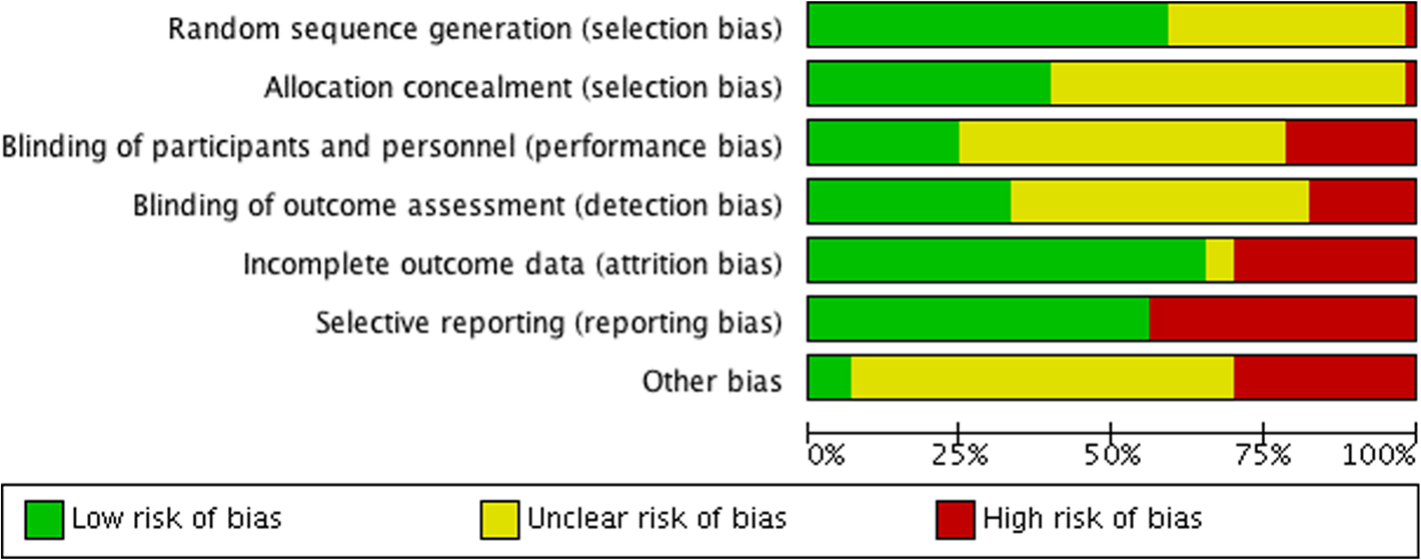
Risk of bias in included studies assessed using the Cochrane tool

Only in two papers (1.9%) were all domains considered as having low RoB, while in 33 (30.8%) there was a low RoB in at least half of them (4/7 domains). In eight studies (7.5%) there was a high RoB in at least half the categories and in 39 studies (36.5%) the risk of bias was unclear (Additional file 3; Fig. 7).

The percentage of trials in the last 5 years that had a low RoB in at least half the domains was higher compared with trials published earlier (33.3% vs 29.7%). However, the percentage of studies high or unclear RoB in at least half the domains in the last 5 years was also higher (high Rob – 9.1% vs 6.8%; unclear Rob – 42.4% vs 33.8%).

Only one study was not randomised. Computer-generated randomisation was the most used mechanism, present in 37.4% of studies (*n* = 40). Regarding the type of randomisation: 23.4% (*n* = 25) used randomisation in blocks, 15% (*n* = 16) stratified, 4.7% (*n* = 5) simple and 0.9% (*n* = 1) used a minimisation method. Most studies used a person, unconnected with the study (e.g., an independent statistical colleague or the pharmacist), to guarantee allocation concealment.

Regarding blinding (of participants, investigators and outcome assessment), 19.6% (*n* = 21) of studies were double-blind, 14% (*n* = 15) single-blind, in 6.5% (*n* = 7) all elements were blinded and 19.6% (*n* = 21) were open-label studies. In 40.2% (*n* = 43) this information was not reported.

According to the instructions of the Cochrane tool, when the primary outcome was not explicit, risk of bias was considered to be high, since it was not clear if the variables were chosen or not based on the results.

## Discussion

This review identified 107 CCTs assessing PC interventions for patients and/or families, the majority of them performed in the USA and the UK. Only one study was not randomized. The amount of missing data is very high in almost all methodological factors evaluated. Overall there is no data from the trial quality appraisal to suggest that reporting of methods is improving.

### Defining “palliative care”: who, what interventions, when?

In our review, we have chosen to accept as a palliative intervention that which the authors assumed to be such. As mentioned before, with increased awareness that non-oncology patients could benefit from a palliative approach, a series of transformations in the concept, interventions and objectives of “palliative care” occurred [1]. This diversity is reflected in the lack of a common lexicon in PC core terms (such as “palliative care” or “end-of-life”) making it not only difficult to ensure that all readers facing the same study reach similar conclusions, but also to define the population and specific interventions of palliative care [14, 26, 27]. Other reviews, such as Lorenz et al. in 2005 review that intended to evaluate the evidence in the field from the perspective of concerns important to patients, caregivers, and the health care system reported the same difficulties, and in 2003 Bausewein et al., in a review on the challenges defining PC, highlighted the lack of clarity in definition and terminology regarding this subject [28, 29].

### Who?

Our results showed that there was a clear predominance of interventions directed to oncological patients (e.g. the comparison of two different methods of therapy administration in the treatment of breakthrough pain in patients with cancer) corresponding to 56.1% of the included studies. If at first this looks normal due to the initial focus of PC on patients dying from cancer, the difference between the percentage of oncology studies and studies of other specialties (56.1% vs. 43.9%) seems to show that we are now beginning to get used to the idea of PC in cancer, but for other diseases this is far from reality. It is also relevant that in 27.1% of the studies dementia was an exclusion criterion. With people living longer and suffering more from diseases that are associated with cognitive impairment, the number of people who are demented and may benefit from PC intervention is increasing. Therefore, cognitive impairment and dementia should not be excluded from the palliative care population, since this can threaten the external validity of studies [30, 31].

### What interventions?

Regarding the type of intervention, the number of studies assessing pharmacological and non-pharmacological interventions was very similar (43.9% vs. 44.9%), with the majority of them evaluating interventions for symptomatic control (70.1%). Other types of interventions, such as non-pharmacologic complementary therapies (7.5%) and home-care based (3.7%), or different aspects of care such as communication (12.1%) or family support (5.6%) were less covered. Albers et al. [26] and Hui et al. [32] in two systematic reviews on methodological issues in PC, pointed out the imbalance between pharmacological interventions and other interventions not related with symptom control, which represented 5% or less of the total RCTs in palliative care. The National Institute of Health in the USA highlighted that few publications on palliative care research reflected the growing needs of patients [33]. Even in the context of symptomatic control research, important gaps in clinical evidence should be addressed. For example nonpain symptoms, such as breathlessness or delirium, are still poorly understood and symptom burden continues to be the main complaint of patients and cause of dropout from studies despite relief of distressing symptoms being considered one of the guiding principles for palliative care practitioners [10, 34].

### When?

In this review the prognosis of patients ranged between “less than one week” to 24 months. The definition of palliative care points towards a population with life-limiting disease and, when cure is not possible, what is frequently understood as the care of patients in their last weeks or days of life [10, 35]. However, some diseases, especially chronic diseases, severely affect the quality of life of patients and family members for many years, this led to considering palliative care earlier, and including in more recent definitions the initiation of palliative care at the time of diagnosis and provided concordantly with all other disease-directed or curative treatments [10].

### The place of RCTs in palliative care research

Of the 939 identified citations, only 11.4% (*n* = 107) were CCTs evaluating palliative care interventions in patients and/or families. This is in line with previous methodological reviews, Hui et al. [5, 32] reported in 2011 that RCTs only comprised 5.6% (*n* = 47) of the studies and in 2006 Kaasa et al. [27], drew attention to the fact that only 4.3% of publications were prospective evaluations of interventions with case series (50.7%, *n* = 462) and cross-sectional studies (17.7%, *n* = 149) being the most common study designs. In 2014, Aoun and Nekolaichuk [9] reported that Cochrane reviews in palliative care failed to provide good evidence because of the few numbers and poor internal and external validity of primary studies. Although these types of studies are a minority in palliative care research, our results show that the number CCTs has increased in recent years.

### Challenges performing RCTs in palliative care

#### Recruitment, attrition and powered samples

The samples of the included studies vary between 9 and 820 participants, with a median of 64.5. It is not uncommon to find statements suggesting that it is unethical to involve people with palliative care needs in research because of their increased vulnerability [27]. Aoun and coworkers [9], in a review about the challenges of evidence-based medicine (EBM) in palliative care research demonstrated precisely the opposite: the participation in experimental protocols was not perceived as an additional stress, but rather like a personal gain in a selfless perspective related with a moderate-to-high benefit. To caregivers, this collaboration is seen as an added value for patients, for themselves, and for future families that need palliative care assistance. Recently, the result of a workshop and consensus exercise (MORECare study), about best practice on ethical concerns in PC research [33], affirmed that it is ethically desirable for patients and their families with palliative care needs to be offered the opportunity to be involved in research and reminded of the existence of relevant international recommendations to overcome some of the ethical challenges faced. Abernethy et al. [36], in a review on key insights to enhance the enrolment in palliative care trials, suggested strategies to successfully recruit patients to large-scale randomised clinical trials, for example where appropriate the adoption of flexible interventions, the reduction of treatment time periods, and the reduction of the number of study assessments including in particular those that are invasive or time consuming.

Our results show a median attrition rate of 22%. A review by Hui et al. [13] found a median attrition rate of 44% in palliative oncological CT. When using a cut-off of ≤ 20% of losses to follow-up and comparing with a review of 71 RCT in four top medical journals showed a dropout rates of ≥ 20% in 18% of the trials [25], we can assume our 40.2% of studies above this cut-off as a high dropout rate. It is unavoidable to have some missing data, but ignoring it is not acceptable, since it represents a significant risk to the power, precision and generalizability of trials results. Looking at our pooled results, there was a higher percentage of dropouts in intervention arm. Hussain et al. [37] in a review on missing data in PC RCT reported a similar result with a high dropout rate in intervention arm. However, as we mentioned in results section there was a high level of heterogeneity between studies that didn’t allow to be conclusive in relation to this question. Furthermore, Bell et al. [25] suggested that for an accurate analysis of attrition bias in pooled results is not enough to know the differential dropout rates, is also necessary take into account the type of missingness (at random, completely at random or not at random), the analysis methods and the effect that is being estimated. The authors suggested the use of mixed models methods as a strategy to estimate unbiased treatment effects, under assumptions regarding the misingness mechanism(s).

Of the 107 included clinical trials, only 53.3% of studies reported a sample size calculation, and of these only 36.8% (*n* = 21) reached the minimum of patients estimated. This is a major problem in clinical research because, as mentioned above, it can be misleading either by missing realistic moderate treatment effects that would be clinically important, or by overestimating the size of a treatment effect and finding it statistically significant purely due to chance [38]. Visser et al. [5] already reported in 2015 that most of the primary studies used in palliative care reviews were methodologically flawed and those that were considered higher quality were inadequately powered.

### Outcomes and assessment tools

Besides the large diversity of study outcomes, our results demonstrate the absence in a significant percentage of studies (40.2%) of an explicit defined primary outcome, which increases the risk of reporting bias since it is not ensured that variables presented were not chosen based on the study results. Because of the great heterogeneity in population and type of interventions, there is still a lack of consensus in palliative care field about the best outcome measures and clinically meaningful differences for each outcome.

In this review only 3.7% of the applied scales are in the list recommended by The National Palliative Care Research Center. The choice of assessment tools is very important in study protocols and one of the challenges of reaching high quality research. Although several instruments can be used to assess outcomes, not all were developed and validated for use in a palliative care population and so not the most appropriate [39].

### The use of placebo-control trials

Our results show that the most used comparator was non-intervention/best supportive care (46.7%, *n* = 50). Best supportive care (BSC) interventions were defined by Jassem et al. (2008) as “treatment administered with the intent to maximize quality of life without a specific antineoplastic regimen” [40]. However, this method has been criticised for poor reporting and for lack of standardisation among trial participants. To overcome these threats, Nipp et al. [41] suggested the use of the published BSC standards and the improvement in reporting the components of BSC control arm [41, 42].

The placebo effect has been shown to be relatively consistent over many studies and has been approximated to account, in general, for up to 35% of the treatment effect [43]. In 1997 two articles [44, 45] with arguments for and against placebo-controlled trials in palliative care were published. The two agree that many of the interventions in palliative care have never been proven to be effective and their use is based on anecdotal reports and/or physician preferences. Hardy et al. (1997), in line with the tenets of evidence-based medicine [43], used this as one of the reasons to encourage the use of placebo-controlled trials and states that when there is no evidence that a drug is better than placebo, and knowing the powerful effect of placebo, there can be no argument against its use [44].

### Quality of reporting analysis

A key finding of this systematic review was the low overall reporting quality of CTs in palliative care and the amount of missing data in trial reporting. Although the number of published studies has increased significantly in the last years, we found no relevant improvement in the overall methodological quality of studies. Only 2/107 papers (1.9%) were evaluated as having a low risk of bias in all domains, and 30.8% (*n* = 33) in at least half of domains. A review of evidence-based practice in palliative care from 2015 [5] supports our findings and attributes the poor quality of trials to the barriers that were already mentioned in this review, such as difficulties in recruitment, in reaching samples with a significant power, and in defining outcomes. In another study from 2014 the authors state that recent reviews in the field continue to report a lack of strong evidence for important topics, due in part to the methodological weaknesses in the existing studies [46].

Allocation concealment and blinding were the aspects poorly reported. Lai [47], in a systematic review of primary treatment in brain tumours, suggested that allocation concealment, blinding, type of analyses and details of randomisation were the poorly reported aspects because of the researcher difficulty in reporting this type data and due to the lack of awareness of the importance of these features. Regarding blinding, in many interventions this method is difficult to apply. However, it is important to use it to minimise bias, especially when the outcome of interest is subjective [48]. The “other bias” domain has the highest percentage of “uncertain risk of bias”. The assessment of this item was based on comparability of trial arms and sample size calculations, but most of all with a global evaluation of methodological flaws and amount of missing information in each trial (Additional file 4). Studies with major gaps in other key methodological characteristics commonly also did not fulfil our criteria for a low risk of other bias.

Quality of a clinical trial is a multidimensional concept that includes study design, conduction, type of analysis, clinical relevance and quality of reporting [49]. In this review we evaluated the quality of reporting, and based on this indirectly inferred studies quality. The focus on the assessment of trial reporting is based on the evidence that studies of lower methodological quality tend to report larger treatment effects than high quality studies [50–52]. It cannot be excluded that obtaining additional information from study protocols or directly from trial investigator could ensure a more accurate assessment of studies quality [9, 53].

Chen et al. [46], in a survey of palliative care researchers about the barriers to improving research in palliative care, highlights as one of the five major identified barriers the lack of research training programs and formal training opportunities, such as research fellowships, which are limiting factors in equipping a researcher workforce. Today, to assist investigators in this field, tools such as the CONSORT statement and SPIRIT, were created to help in the reporting of study results and to raise the quality of studies [18, 54].

### Shortcomings

Although the current study had included a pre-specified search strategy and two independent investigators performed the selection, we were restricted to one database (MEDLINE), which can not ensure we have identified all studies that meet the eligibility criteria. As mentioned above, there is a large variability in palliative care terminology. For that reason the identification of clinical trials in this review was made, not according to a specific and strict definition, but in line with author’s assumption of what constitutes a palliative care intervention. As a result, the number of retrieved citations was high, with an elevated percentage of manuscripts that did not meet inclusion criteria for clinical trials. Of the 939 identified citations, 107 met the inclusion criteria and were analysed. Compared with previous reviews, our search strategy generated the largest list of clinical trials concerning therapeutic interventions in palliative care appraised and we believe that our results are representative of interventional studies in PC.

## Conclusions

With palliative care research becoming increasingly necessary it is not enough to conduct more studies, it is also necessary to improve the quality of evidence.

Palliative care research is trying to deal with the large heterogeneity, the ethical and methodological issues resulting from the expansion of its scope of intervention, while at the same time it still seems too tied to the concept of terminal care of oncological patients, with a level of quality of reporting that has not evolved.

To our knowledge, this is the most comprehensive attempt to review clinical trials in palliative care literature. According to our results, it seems that the first step in generating not just valid, but also generalisable knowledge, is to clearly define palliative care populations, types of intervention and time to referral, establishing a common lexicon for clinicians and researchers. This will allow consensus to be achieved on the best outcomes and clinically meaningful differences, and will facilitate the choice of study design as well as promoting strategies to bypass the major barriers in palliative care research. The use of tools to help reporting study outcomes, such as CONSORT or SPIRIT, could also be a simple and efficient way of improving the quality of studies.

## Additional files

Additional file 1: Research strategy description. (DOCX 14 kb)
Additional file 2: References of included studies. (DOCX 29 kb)
Additional file 3: Risk of bias assessment evaluation. (DOCX 20 kb)
Additional file 4: Criteria for judging “other risk of bias” in the Cochrane Risk of bias assessment tool. (DOCX 57 kb)

## Abbreviations

BSC: Best supportive care
CCT: Control clinical trial
CI: Confidence interval
EBM: Evidence-based medicine
IF: Impact factor
OR: Odds ratio
RCT: Randomised controlled trial
RoB: Risk of bias
SD: Standard deviation
