# Supplementary material for: Clinical trials in palliative care: a systematic review of their methodological characteristics and of the quality of their reporting
Source: BMC Palliat Care. 2017 Jan 25;16:10. doi: 10.1186/s12904-016-0181-9 (PMC5264484; doi:10.1186/s12904-016-0181-9)
Supplement: Additional file 1: — Research strategy description. (DOCX 14 kb) [file 12904_2016_181_MOESM1_ESM.docx]

**Web appendix 1**

Research strategy description

Database: Ovid MEDLINE(R) In-Process & Other Non-Indexed Citations and Ovid MEDLINE(R) <1946 to Present>

Search Strategy:

--------------------------------------------------------------------------------

1 (palliative adj3 (care or caring or ill*)).ab,ti. (16101)

2 randomized controlled trial.pt. (384788)

3 controlled clinical trial.pt. (88618)

4 randomized.ab. (309487)

5 placebo.ab. (158176)

6 clinical trials as topic.sh. (170815)

7 randomly.ab. (224178)

8 trial.ti. (133051)

9 non?randomized.ab,ti. (8500)

10 2 or 3 or 4 or 5 or 6 or 7 or 8 or 9 (941361)

11 exp animals/ not humans.sh. (3986356)

12 10 not 11 (868454)

13 1 and 12 (939)
