# Supplementary material for: Clinical trials in palliative care: a systematic review of their methodological characteristics and of the quality of their reporting
Source: BMC Palliat Care. 2017 Jan 25;16:10. doi: 10.1186/s12904-016-0181-9 (PMC5264484; doi:10.1186/s12904-016-0181-9)
Supplement: Additional file 2: — References of included studies. (DOCX 29 kb) [file 12904_2016_181_MOESM2_ESM.docx]

**Web appendix 2**

References of included studies

1. Ahmedzai S, Brooks D. Transdermal fentanyl versussustained-release oral morphine in cancer pain: Preference, efficacy, and quality of life. *J Pain Symptom Manag.* 1997;13(5):254-261. doi:10.1016/S0885-3924(97)00082-1.

2. Ahronheim JC, Morrison RS, Morris J, Baskin S, Meier DE. Palliative care in advanced dementia: a randomized controlled trial and descriptive analysis. *J Palliat Med* 2000;3(3):265-73. doi:10.1089/jpm.2000.3.265.

3. Allen RS, Harris GM, Burgio LD, et al. Can Senior Volunteers Deliver Reminiscence and Creative Activity Interventions ? Results of the Legacy Intervention Family Enactment Randomized Controlled Trial. *J Pain Symptom Manag.* 2014;48(4):590-601. doi:10.1016/j.jpainsymman.2013.11.012.

4. Auret KA, Schug SA, Bremner AP, Bulsara M. A randomized, double-blind, placebo-controlled trial assessing the impact of dexamphetamine on fatigue in patients with advanced cancer. *J Pain Symptom Manag.* 2009;37(4):613-21. doi:10.1016/j.jpainsymman.2008.03.016.

5. Badr H, Smith CB, Goldstein NE, Gomez JE. Dyadic Psychosocial Intervention for Advanced Lung Cancer Patients and Their Family Caregivers : Results of a Randomized Pilot Trial. *Cancer* 2014. doi:10.1002/cncr.29009.

6. Bakitas M, Lyons KD, Hegel MT, et al. The project ENABLE II randomized controlled trial to improve palliative care for rural patients with advanced cancer: baseline findings, methodological challenges, and solutions. *Palliat Support Care* 2009;7(1):75-86. doi:10.1017/S1478951509000108.

7. Bennett MI, Johnson MI, Brown SR, Radford H, Brown JM, Searle RD. Feasibility study of Transcutaneous Electrical Nerve Stimulation (TENS) for cancer bone pain. *J Pain* 2010;11(4):351-9. doi:10.1016/j.jpain.2009.08.002.

8. Bots CP, Brand HS, Veerman ECI, et al. The management of xerostomia in patients on haemodialysis: comparison of artificial saliva and chewing gum. *Palliat Med* 2005;19:202-207. doi:10.1191/0269216305pm1009oa.

9. Brandsta M, Ko M, Borasio GD, Fensterer V, Fegg MJ. Mindfulness in informal caregivers of palliative patients. *Palliat Support Care* 2015:11-18. doi:10.1017/S1478951513000400.

10. Brännström M, Boman K. Effects of person-centred and integrated chronic heart failure and palliative home care . PREFER : a randomized controlled study. *Eur J Hear. Fail* 2014:42-51.

11. Bruera E, Willey J, Cohen M, Palmer JL. Expressive writing in patients receiving palliative care: a feasibility study. *J Palliat Med* 2008;11(1):15-9. doi:10.1089/jpm.2007.0112.

12. Bruera E, Sloan P, Mount B, Scott J, Suarez-Almazor M. A Randomized, Double-Blind, Double-Dummy, Crossover Trail Comparing the Safety and Efficacy of Oral Sustained-Release Hydromorphone With Immediate-Release Hydromorphone in Patients With Cancer Pain.pdf. *J Clin Oncol* 1996:1713-17.

13. Bruera E, Palmer JL, Bosnjak S, et al. Methadone versus morphine as a first-line strong opioid for cancer pain: A randomized, double-blind study. *J Clin Oncol* 2004;22(1):185-192. doi:10.1200/JCO.2004.03.172.

14. Bruera E, Valero V, Driver L, et al. Patient-controlled methylphenidate for cancer fatigue: a double-blind, randomized, placebo-controlled trial. *J Clin Oncol* 2006;24(13):2073-8. doi:10.1200/JCO.2005.02.8506.

15. Brumley R, Enguidanos S, Jamison P, et al. Increased satisfaction with care and lower costs: results of a randomized trial of in-home palliative care. *J Am Geriatr Soc* 2007;55(7):993-1000. doi:10.1111/j.1532-5415.2007.01234.x.

16. Charles MA, Reymond L, Israel F. Relief of incident dyspnea in palliative cancer patients: a pilot, randomized, controlled trial comparing nebulized hydromorphone, systemic hydromorphone, and nebulized saline. *J Pain Symptom Manag.* 2008;36(1):29-38. doi:10.1016/j.jpainsymman.2007.08.016.

17. Cherin D, Huba G. Evaluation of the transprofessional model of home health care for HIV/AIDS. *Home Heal. Care Serv Q* 1998;17(1):55-72. doi:10.1300/J027v17n01.

18. Cheung W, Aggarwal G. Palliative care teams in the intensive care unit: a randomised, controlled, feasibility study. *Crit Care Resusc* 2010;12(1):28-35.

19. Chochinov HM, Kristjanson LJ, Breitbart W, et al. Effect of dignity therapy on distress and end-of-life experience in terminally ill patients: a randomised controlled trial. *Lancet Oncol* 2011;12(8):753-62. doi:10.1016/S1470-2045(11)70153-X.

20. Clayton JM, Butow PN, Tattersall MHN, et al. Randomized controlled trial of a prompt list to help advanced cancer patients and their caregivers to ask questions about prognosis and end-of-life care. *J Clin Oncol* 2007;25(6):715-23. doi:10.1200/JCO.2006.06.7827.

21. Cornbleet M, Campbell P. Patient-held records in cancer and palliative care: a randomized, prospective trial. *Palliat Med* 2002;16:205-212.

22. Corner J, Plant H, Hern RA, Bailey C. Non-pharmacological intervention for breathlessness in lung cancer. *Palliat Med* 1996;10. doi:10.1177/026921639601000405.

23. Currow D. Comparison of metal Versus Vialon subcutaneous catheters in a palliative care setting. *Palliat Med* 1994;8:333-336.

24. Dreher M, Storre JH, Windisch W. Noninvasive ventilation during walking in patients with severe COPD: a randomised cross-over trial. *Eur Respir J* 2007;29(5):930-6. doi:10.1183/09031936.00075806.

25. Duggleby WD, Degner L, Williams A, et al. Living with hope: initial evaluation of a psychosocial hope intervention for older palliative home care patients. *J Pain Symptom Manag.* 2007;33(3):247-57. doi:10.1016/j.jpainsymman.2006.09.013.

26. Dyar S, Lesperance M, Shannon R, Sloan J, Colon-Otero G. A nurse practitioner directed intervention improves the quality of life of patients with metastatic cancer: results of a randomized pilot study. *J Palliat Med* 2012;15(8):890-5. doi:10.1089/jpm.2012.0014.

27. Edmonds P, Hart S, Wei Gao, et al. Palliative care for people severely affected by multiple sclerosis: evaluation of a novel palliative care service. *Mult Scler* 2010;16(5):627-36. doi:10.1177/1352458510364632.

28. Evangelista LS, Lombardo D, Malik S, Ballard-Hernandez J, Motie M, Liao S. Examining the effects of an outpatient palliative care consultation on symptom burden, depression, and quality of life in patients with symptomatic heart failure. *J Card Fail* 2012;18(12):894-899. doi:10.1016/j.cardfail.2012.10.019.

29. Farquhar MC, Prevost AT, Mccrone P, et al. Is a specialist breathlessness service more effective and cost-effective for patients with advanced cancer and their carers than standard care ? Findings of a mixed-method randomised controlled trial. *BMC Med* 2014:1-13. doi:10.1186/s12916-014-0194-2.

30. Flock P. Pilot Study to Determine the Effectiveness of Diamorphine Gel to Control Pressure Ulcer Pain. *J Pain Symptom Manag.* 2003;25(6):547-554. doi:10.1016/S0885-3924(03)00140-4.

31. Galfin JM, Watkins ER, Harlow T. A brief guided self-help intervention for psychological distress in palliative care patients: a randomised controlled trial. *Palliat Med* 2012;26(3):197-205. doi:10.1177/0269216311414757.

32. Gammaitoni a R, Gallagher RM, Welz M, Gracely EJ, Knowlton CH, Voltis-Thomas O. Palliative pharmaceutical care: a randomized, prospective study of telephone-based prescription and medication counseling services for treating chronic pain. *Pain Med* 2000;1(4):317-31.

33. Ganz PA, Figlin RA, Haskell CM. Supportive Care versus Supportive Care and Combination Chemotherapy in Metastic Non-Small Cell Lung Cancer. *Cancer* 1989;63:1271-78.

34. Grande GE, Todd C, Barclay S, Farquhar M. Does hospital at home for palliative care facilitate death at home? Randomised controlled trial. *BMJ* 1999;319:1472-75.

35. Grimbert D, Lubin O. Dyspnée et aérosols de morphine dans les soins palliatifs du cancer broncho-pulmonaire. *Rev Mal Respir* 2004;21:1091-1097.

36. Gutgsell KJ, Schluchter M, Margevicius S, et al. Music therapy reduces pain in palliative care patients: a randomized controlled trial. *J Pain Symptom Manag.* 2013;45(5):822-31. doi:10.1016/j.jpainsymman.2012.05.008.

37. Hall S, Goddard C, Opio D, Speck P, Higginson IJ. Feasibility, acceptability and potential effectiveness of Dignity Therapy for older people in care homes: a phase II randomized controlled trial of a brief palliative care psychotherapy. *Palliat Med* 2012;26(5):703-12. doi:10.1177/0269216311418145.

38. Hardy J, Ling J, Mansi J, et al. Pitfalls in placebo-controlled trials in palliative care: dexamethasone for the palliation of malignant bowel obstruction. *Palliat Med* 1998;12(6):437-42. doi:10.1191/026921698666334766.

39. Higginson IJ, Bausewein C, Reilly CC, et al. An integrated palliative and respiratory care service for patients with advanced disease and refractory breathlessness: a randomised controlled trial. *Lancet Respir Med* 2014;2(12):979-87. doi:10.1016/S2213-2600(14)70226-7.

40. Homs MY V, Essink-Bot M-L, Borsboom GJJM, Steyerberg EW, Siersema PD. Quality of life after palliative treatment for oesophageal carcinoma -- a prospective comparison between stent placement and single dose brachytherapy. *Eur J Cancer* 2004;40:1862-1871. doi:10.1016/j.ejca.2004.04.021.

41. Hopkinson JB, Fenlon DR, Okamoto I, et al. The deliverability, acceptability, and perceived effect of the Macmillan approach to weight loss and eating difficulties: a phase II, cluster-randomized, exploratory trial of a psychosocial intervention for weight- and eating-related distress in people wi. *J Pain Symptom Manag.* 2010;40(5):684-95. doi:10.1016/j.jpainsymman.2010.02.015.

42. Horne-Thompson A, Grocke D. The effect of music therapy on anxiety in patients who are terminally ill. *J Palliat Med* 2008;11(4):582-90. doi:10.1089/jpm.2007.0193.

43. Hudson PL, Aranda S, Hayman-White K. A psycho-educational intervention for family caregivers of patients receiving palliative care: a randomized controlled trial. *J Pain Symptom Manag.* 2005;30(4):329-41. doi:10.1016/j.jpainsymman.2005.04.006.

44. Hudson P, Trauer T, Kelly B, et al. Reducing the psychological distress of family caregivers of home-based palliative care patients: short-term effects from a randomised controlled trial. *Psychooncology* 2013;22(9):1987-93. doi:10.1002/pon.3242.

45. Israel FJ, Parker G, Charles M, Reymond L. Lack of Benefit From Paracetamol (Acetaminophen) for Palliative Cancer Patients Requiring High-Dose Strong Opioids: A Randomized, Double-Blind, Placebo-Controlled, Crossover Trial. *J Pain Symptom Manag.* 2010;39(3):548-554. doi:10.1016/j.jpainsymman.2009.07.008.

46. Jordhøy MS, Fayers P, Saltnes T, Ahlner-Elmqvist M, Jannert M, Kaasa S. A palliative-care intervention and death at home: a cluster randomised trial. *Lancet* 2000;356(9233):888-893. doi:10.1016/S0140-6736(00)02678-7.

47. Julião M, Oliveira F, Nunes B, Vaz Carneiro A, Barbosa A. Efficacy of dignity therapy on depression and anxiety in Portuguese terminally ill patients: a phase II randomized controlled trial. *J Palliat Med* 2014;17(6):688-95. doi:10.1089/jpm.2013.0567.

48. Kamboj SK, Conroy L, Tookman A, Carroll E, Jones L, Curran H V. Effects of immediate-release opioid on memory functioning : a randomized-controlled study in patients receiving sustained-release opioids. *Eur J Pain* 2014;18:1376-1384. doi:10.1002/j.1532-2149.2014.498.x.

49. Kissane DW, McKenzie M, Bloch S, Moskowitz C, McKenzie DP, O’Neill I. Family focused grief therapy: a randomized, controlled trial in palliative care and bereavement. *Am J Psychiatry* 2006;163(7):1208-18. doi:10.1176/appi.ajp.163.7.1208.

50. Kress HG, Orońska A, Kaczmarek Z, Kaasa S, Colberg T, Nolte T. Efficacy and tolerability of intranasal fentanyl spray 50 to 200 microg for breakthrough pain in patients with cancer: a phase III, multinational, randomized, double-blind, placebo-controlled, crossover trial with a 10-month, open-label extension treatmen. *Clin Ther* 2009;31(6):1177-91. doi:10.1016/j.clinthera.2009.05.022.

51. Kutner J, Smith M. Massage therapy versus simple touch to improve pain and mood in patients with advanced cancera randomized trial. *Ann Intern Med* 2008;149(6):369-379.

52. Kyle G. Evaluating the effectiveness of aromatherapy in reducing levels of anxiety in palliative care patients: Results of a pilot study. *Complement Ther Clin Pr.* 2006;12:148-155. doi:10.1016/j.ctcp.2005.11.003.

53. Latimer EJ, Crabb MR, Roberts JG, Ewen M, Roberts J. The Patient Care Travelling Record© in Palliative Care. *J Pain Symptom Manag.* 1998;16(1):41-51. doi:10.1016/S0885-3924(98)00027-X.

54. Laval G, Girardier J, Lassauniere J. The use of steroids in the management of inoperable intestinal obstruction in terminal cancer patients: do they remove the obstruction? *Palliat Med* 2000;14:3-10.

55. Laval G, Rousselot H, Toussaint-Martel S, et al. SALTO: a randomized, multicenter study assessing octreotide LAR in inoperable bowel obstruction. *Bull Cancer* 2012;99(2):E1-9. doi:10.1684/bdc.2011.1535.

56. LeCaer H, Greillier L, Corre R, et al. A multicenter phase II randomized trial of gemcitabine followed by erlotinib at progression, versus the reverse sequence, in vulnerable elderly patients with advanced non small-cell lung cancer selected with a comprehensive geriatric assessment (the GFPC . *Lung Cancer* 2012;77:97-103. doi:10.1016/j.lungcan.2012.02.004.

57. Lee C, Vather R, O’Callaghan A, et al. Validation of the phase II feasibility study in a palliative care setting: gastrografin in malignant bowel obstruction. *Am J Hosp Palliat Care* 2012;30(8):752-8. doi:10.1177/1049909112471422.

58. Lim JTW, Wong ET, Aung SKH. Is there a role for acupuncture in the symptom management of patients receiving palliative care for cancer? A pilot study of 20 patients comparing acupuncture with nurse-led supportive care. *Acupunct Med* 2011;29(3):173-9. doi:10.1136/aim.2011.004044.

59. Lindholm E, Daneryd P, Körner U. Effects of recombinant erythropoietin in palliative treatment of unselected cancer patients. *Clin Cancer Res* 2004;10:6855-6864.

60. Link KH, Pillasch J, Formentini A, et al. Downstaging by regional chemotherapy of non-resectable isolated colorectal liver metastases. *Eur J Surg Oncol* 1999;25:381-388. doi:10.1053/ejso.1999.0661.

61. Lundholm K, Körner U, Gunnebo L, et al. Insulin treatment in cancer cachexia: effects on survival, metabolism, and physical functioning. *Clin Cancer Res* 2007;13(9):2699-706. doi:10.1158/1078-0432.CCR-06-2720.

62. Matlock DD, Keech T a E, McKenzie MB, Bronsert MR, Nowels CT, Kutner JS. Feasibility and acceptability of a decision aid designed for people facing advanced or terminal illness: a pilot randomized trial. *Heal. Expect* 2011;17(1):49-59. doi:10.1111/j.1369-7625.2011.00732.x.

63. Menahem S, Shvartzman P. Continuous subcutaneous delivery of medications for home care palliative patients-using an infusion set or a pump? *Support Care Cancer* 2010;18:1165-1170. doi:10.1007/s00520-009-0736-x.

64. Miller D, Chibnall J. Supportive-affective group experience for persons with life-threatening illness: reducing spiritual, psychological, and death-related distress in dying patients. *J Palliat Med* 2005;8(2):333-343.

65. Mills ME, Murray LJ, Johnston BT, Cardwell C, Donnelly M. Does a patient-held quality-of-life diary benefit patients with inoperable lung cancer? *J Clin Oncol* 2009;27(1):70-7. doi:10.1200/JCO.2008.17.5687.

66. Mystakidou K, Katsouda E, Kouloulias V, Kouvaris J, Tsiatas M, Vlahos L. Comparison of transdermal fentanyl with codeine/paracetamol, in combination with radiotherapy, for the management of metastatic bone pain. *J Opioid Manag* 2005;1(4):204-210.

67. Mok E, Lau K, Lai T, Ching S. The meaning of life intervention for patients with advanced-stage cancer: development and pilot study. *Oncol Nurs Forum* 2012;39(6):E480-8. doi:10.1188/12.ONF.E480-E488.

68. Nava S, Ferrer M, Esquinas A, et al. Palliative use of non-invasive ventilation in end-of-life patients with solid tumours: A randomised feasibility trial. *Lancet Oncol* 2013;14:219-227. doi:10.1016/S1470-2045(13)70009-3.

69. Philip J, Gold M, Milner A, Di Iulio J, Miller B, Spruyt O. A randomized, double-blind, crossover trial of the effect of oxygen on dyspnea in patients with advanced cancer. *J Pain Symptom Manag.* 2006;32(6):541-50. doi:10.1016/j.jpainsymman.2006.06.009.

70. Popa-Velea O, Cernat B, Tambu A. Influence of personalized therapeutic approach on quality of life and psychiatric comorbidity in patients with advanced colonic cancer requiring palliative care. *J Med Life* 2010;3(3):343-7.

71. Prentice WM, Roth LJ, Kelly P. Topical benzydamine cream and the relief of pressure pain. *Palliat Med* 2004;18(6):520-4.

72. Rabow MW, Dibble SL, Pantilat SZ, McPhee SJ. The comprehensive care team: a controlled trial of outpatient palliative medicine consultation. *Arch Intern Med* 2004;164(1):83-91. doi:10.1001/archinte.164.1.83.

73. Ramesh PR, Kumar KS, Rajagopal MR, Balachandran P, Warrier PK. Managing morphine-induced constipation: A controlled comparison of an Ayurvedic formulation and senna. *J Pain Symptom Manag.* 1998;16(4):240-244. doi:10.1016/S0885-3924(98)00080-3.

74. Ranson M, Davidson N, Nicolson M, et al. Randomized trial of paclitaxel plus supportive care versus supportive care for patients with advanced non-small-cell lung cancer. *J Natl Cancer Inst* 2000;92(13):1074-80.

75. Reck M, von Pawel J, Macha HN, et al. Efficient palliation in patients with small-cell lung cancer by a combination of paclitaxel, etoposide and carboplatin: Quality of life and 6-years’-follow-up results from a randomised phase III trial. *Lung Cancer* 2006;53:67-75. doi:10.1016/j.lungcan.2006.04.001.

76. Reinhardt JP, Chichin E, Posner L, Kassabian S. Vital conversations with family in the nursing home: preparation for end-stage dementia care. *J Soc Work End Life Palliat Care* 2014;10(2):112-26. doi:10.1080/15524256.2014.906371.

77. Reymond L, Charles M a, Bowman J, Treston P. The effect of dexamethasone on the longevity of syringe driver subcutaneous sites in palliative care patients. *Med J Aust* 2003;178(10):486-9.

78. Salas S, Frasca M, Planchet-Barraud B, et al. Ketamine analgesic effect by continuous intravenous infusion in refractory cancer pain: considerations about the clinical research in palliative care. *J Palliat Med* 2012;15(3):287-93. doi:10.1089/jpm.2011.0353.

79. Sampson EL, Jones L, Thuné-Boyle IC V, et al. Palliative assessment and advance care planning in severe dementia: an exploratory randomized controlled trial of a complex intervention. *Palliat Med* 2010;25(3):197-209. doi:10.1177/0269216310391691.

80. Schofield P. A pilot study into the use of a multi sensory enviorenment (Snoezelen) within a palliative day-care setting. *Int J Palliat Nurs* 2003;9(3):124-9.

81. Sidebottom AC, Jorgenson A, Richards H. Inpatient Palliative Care for Patients with Acute Heart Failure : Outcomes from a Randomized Trial. *J Palliat Med* 2015;18(2):134-142. doi:10.1089/jpm.2014.0192.

82. Slatkin N, Thomas J, Lipman AG, et al. Methylnaltrexone for Treatment of Opioid-Induced Constipation in Advanced Illness Patients. *J Support Oncol* 2009;7(1):39-46.

83. Soden K, Vincent K, Craske S, Lucas C, Ashley S. A randomized controlled trial of aromatherapy massage in a hospice setting. *Palliat Med* 2004;18(2):87-92.

84. Steinhauser KE, Alexander SC, Byock IR, George LK, Olsen MK, Tulsky J a. Do preparation and life completion discussions improve functioning and quality of life in seriously ill patients? Pilot randomized control trial. *J Palliat Med* 2008;11(9):1234-40. doi:10.1089/jpm.2008.0078.

85. Suh S-Y, Choi YS, Oh SC, et al. Caffeine as an adjuvant therapy to opioids in cancer pain: a randomized, double-blind, placebo-controlled trial. *J Pain Symptom Manag.* 2013;46(4):474-82. doi:10.1016/j.jpainsymman.2012.10.232.

86. Zimmermann C, Swami N, Krzyzanowska M, et al. Early palliative care for patients with advanced cancer: a cluster-randomised controlled trial. *Lancet* 2014;383(9930):1721-30. doi:10.1016/S0140-6736(13)62416-2.

87. Temel JS, Greer J a, Muzikansky A, et al. Early palliative care for patients with metastatic non-small-cell lung cancer. *N Engl J Med* 2010;363(8):733-42. doi:10.1056/NEJMoa1000678.

88. Thomas JR, Wallace MS, Yocum RC, Vaughn DE, Haller MF, Flament J. The INFUSE-Morphine study: use of recombinant human hyaluronidase (rHuPH20) to enhance the absorption of subcutaneously administered morphine in patients with advanced illness. *J Pain Symptom Manag.* 2009;38(5):663-72. doi:10.1016/j.jpainsymman.2009.03.009.

89. Todd J, Medicine P, Hospice T. An assessment of the efficacy and tolerability of a ` double dose ’ of normal-release morphine sulphate at bedtime. *Palliat Med* 2002:507-513.

90. Toscani F, Piva L, Corli O, et al. Ketorolac versus Diclofenac Sodium in Cancer Pain. *Arzneimittelforschung* 1994;44(1):550-554.

91. Uitdehaag MJ, van der Velden L-A, de Boer MF, et al. Recordings of consultations are beneficial in the transition from curative to palliative cancer care: a pilot-study in patients with oesophageal or head and neck cancer. *Eur J Oncol Nurs* 2012;16(2):109-14. doi:10.1016/j.ejon.2011.04.006.

92. Varela YA, Sacristán A, González M, Ferrari M, Portugués A, Calvo MJ. Efficacy of senna versus lactulose in terminal cancer patients treated with opioids. *J Pain Symptom Manag.* 1998;15(l):1-7. doi:10.1016/S0885-3924(97)00276-5.

93. Ventafridda V, Toscani F, Tamburini M, et al. Sodium naproxen versus sodium diclofenac in cancer pain control. *Arzneimittelforschung* 1990;40(10):1132-4.

94. Vogel RI, Petzel S V, Cragg J, et al. Development and pilot of an advance care planning website for women with ovarian cancer: a randomized controlled trial. *Gynecol Oncol* 2013;131(2):430-6. doi:10.1016/j.ygyno.2013.08.017.

95. Watanabe S, Pereira J, Tarumi Y, Hanson J, Sc M, Bruera E. A Randomized Double-Blind Crossover Comparison of Continuous and Intermittent Subcutaneous Administration of Opioid for Cancer Pain. *J Palliat Med* 2008;11(4). doi:10.1089/jpm.2007.0176.

96. Cerchietti L, Navigante A, Sauri A, Palazzo F. Hypodermoclysis for control of dehydration in terminal-stage cancer. *Int J Palliat Nurs* 2000;6(8):370-4. doi:10.12968/ijpn.2000.6.8.9060.

97. Cullen MH, Billingham LJ, Woodroffe CM, et al. Mitomycin, ifosfamide, and cisplatin in unresectable non-small-cell lung cancer: effects on survival and quality of life. *J Clin Oncol* 1999;17(10):3188-94.

98. Ducloux D, Guisado H, Pautex S. Promoting sleep for hospitalized patients with advanced cancer with relaxation therapy: experience of a randomized study. *Am J Hosp Palliat Care* 2013;30(6):536-40. doi:10.1177/1049909112459367.

99. Giasson M, Bouchard L. Effect of therapeutic touch on the well-being of persons with terminal cancer. *J. Holist. Nurs.* 1998;16(3):383-98.

100. Hall S, Goddard C, Opio D, Speck PW, Martin P, Higginson IJ. A novel approach to enhancing hope in patients with advanced cancer: a randomised phase II trial of dignity therapy. *BMJ Support Palliat Care* 2011;1(3):315-21. doi:10.1136/bmjspcare-2011-000054.

101. Hansen MJ, Enright RD, Baskin TW, Klatt J. A palliative care intervention in forgiveness therapy for elderly terminally ill cancer patients. *J Palliat Care* 2009;25(1):51-60.

102. Hilliard RE. The effects of music therapy on the quality and length of life of people diagnosed with terminal cancer. *J Music Ther* 2003;40(2):113-37.

103. Tsai P-S, Chen P-L, Lai Y-L, Lee M-B, Lin C-C. Effects of electromyography biofeedback-assisted relaxation on pain in patients with advanced cancer in a palliative care unit. *Cancer Nurs* 30(5):347-53. doi:10.1097/01.NCC.0000290805.38335.7b.

104. Tse MMY, Wong ACF, Ng HN, Lee HY, Chong MH, Leung WY. The effect of a pain management program on patients with cancer pain. *Cancer Nurs* 35(6):438-46. doi:10.1097/NCC.0b013e3182360730.

105. Weber C, Merminod T, Herrmann FR, Zulian GB. Prophylactic anti-coagulation in cancer palliative care: a prospective randomised study. *Support Care Cancer* 2008;16(7):847-52. doi:10.1007/s00520-007-0339-3.

106. Wilcock A, Manderson C, Weller R. Does aromatherapy massage benefit patients with cancer attending a specialist palliative care day centre? *Palliat Med* 2004;18:287-291.

107. Wu J-J, Cui Y, Yang Y-S, et al. Modulatory effects of aromatherapy massage intervention on electroencephalogram, psychological assessments, salivary cortisol and plasma brain-derived neurotrophic factor. *Complement Ther Med* 2014;22(3):456-62. doi:10.1016/j.ctim.2014.04.001.
