# Supplementary material for: Clinical trials in palliative care: a systematic review of their methodological characteristics and of the quality of their reporting
Source: BMC Palliat Care. 2017 Jan 25;16:10. doi: 10.1186/s12904-016-0181-9 (PMC5264484; doi:10.1186/s12904-016-0181-9)
Supplement: Additional file 3: — Risk of bias assessment evaluation. (DOCX 20 kb) [file 12904_2016_181_MOESM3_ESM.docx]

**Web Appendix 3**

Risk of bias assessment of included studies

| **Author, year** | **Randomization** | **Allocation Concealment** | **Blinding of participants and personnel** | **Blinding of outcome assessment** | **Incomplete outcome data adressed?** | **Selective reporting** | **Other bias** |
| --- | --- | --- | --- | --- | --- | --- | --- |
| Ahmedzai, 1997 | Uncertain Risk of Bias | Uncertain Risk of Bias | High Risk of Bias | High Risk of Bias | Low Risk of Bias | Low Risk of Bias | High Risk of Bias |
| Ahronheim, 2000 | Uncertain Risk of Bias | Uncertain Risk of Bias | Uncertain Risk of Bias | Low Risk of Bias | Low Risk of Bias | Low Risk of Bias | Low Risk of Bias |
| Allen, 2014 | Low Risk of Bias | Uncertain Risk of Bias | Uncertain Risk of Bias | Low Risk of Bias | Low Risk of Bias | High Risk of Bias | High Risk of Bias |
| Auret, 2009 | Uncertain Risk of Bias | Low Risk of Bias | Low Risk of Bias | Low Risk of Bias | Low Risk of Bias | High Risk of Bias | Low Risk of Bias |
| Badr, 2014 | Uncertain Risk of Bias | Uncertain Risk of Bias | Uncertain Risk of Bias | Uncertain Risk of Bias | Low Risk of Bias | Low Risk of Bias | High Risk of Bias |
| Bakitas, 2009 | Low Risk of Bias | Low Risk of Bias | High Risk of Bias | Uncertain Risk of Bias | Low Risk of Bias | Low Risk of Bias | Low Risk of Bias |
| Bennett, 2010 | Low Risk of Bias | Low Risk of Bias | Low Risk of Bias | Low Risk of Bias | Low Risk of Bias | Low Risk of Bias | Uncertain Risk of Bias |
| Bots, 2005 | Uncertain Risk of Bias | Uncertain Risk of Bias | Uncertain Risk of Bias | Uncertain Risk of Bias | High Risk of Bias | High Risk of Bias | Uncertain Risk of Bias |
| Brandsta, 2015 | Uncertain Risk of Bias | Uncertain Risk of Bias | Uncertain Risk of Bias | Uncertain Risk of Bias | Low Risk of Bias | High Risk of Bias | High Risk of Bias |
| Brännström, 2014 | Uncertain Risk of Bias | Uncertain Risk of Bias | High Risk of Bias | High Risk of Bias | Low Risk of Bias | High Risk of Bias | High Risk of Bias |
| Bruera, 1996 | Uncertain Risk of Bias | Uncertain Risk of Bias | Low Risk of Bias | Low Risk of Bias | High Risk of Bias | High Risk of Bias | High Risk of Bias |
| Bruera, 2004 | Low Risk of Bias | Low Risk of Bias | Low Risk of Bias | Low Risk of Bias | High Risk of Bias | Low Risk of Bias | Low Risk of Bias |
| Bruera, 2006 | Low Risk of Bias | Low Risk of Bias | Low Risk of Bias | Low Risk of Bias | Low Risk of Bias | Low Risk of Bias | High Risk of Bias |
| Bruera, 2008 | Uncertain Risk of Bias | Uncertain Risk of Bias | Uncertain Risk of Bias | Uncertain Risk of Bias | High Risk of Bias | High Risk of Bias | High Risk of Bias |
| Brumley, 2007 | Low Risk of Bias | Low Risk of Bias | Low Risk of Bias | Uncertain Risk of Bias | Low Risk of Bias | High Risk of Bias | Uncertain Risk of Bias |
| Cerchietti, 2009 | Uncertain Risk of Bias | Uncertain Risk of Bias | Uncertain Risk of Bias | Uncertain Risk of Bias | Low Risk of Bias | Low Risk of Bias | Uncertain Risk of Bias |
| Charles, 2008 | Low Risk of Bias | Low Risk of Bias | Uncertain Risk of Bias | Uncertain Risk of Bias | Low Risk of Bias | Low Risk of Bias | High Risk of Bias |
| Cherin, 1998 | Low Risk of Bias | High Risk of Bias | Uncertain Risk of Bias | Uncertain Risk of Bias | Unclear | High Risk of Bias | Uncertain Risk of Bias |
| Cheung, 2010 | Low Risk of Bias | Uncertain Risk of Bias | High Risk of Bias | High Risk of Bias | Low Risk of Bias | Low Risk of Bias | Uncertain Risk of Bias |
| Chochinov, 2011 | Low Risk of Bias | Low Risk of Bias | Uncertain Risk of Bias | Low Risk of Bias | High Risk of Bias | Low Risk of Bias | Uncertain Risk of Bias |
| Clayton, 2007 | Low Risk of Bias | Low Risk of Bias | High Risk of Bias | High Risk of Bias | Low Risk of Bias | Low Risk of Bias | High Risk of Bias |
| Cornbleet, 2002 | Uncertain Risk of Bias | Uncertain Risk of Bias | Uncertain Risk of Bias | Uncertain Risk of Bias | Low Risk of Bias | Low Risk of Bias | Uncertain Risk of Bias |
| Corner, 1996 | Uncertain Risk of Bias | Uncertain Risk of Bias | Uncertain Risk of Bias | Uncertain Risk of Bias | Low Risk of Bias | High Risk of Bias | High Risk of Bias |
| Cullen, 2003 | Uncertain Risk of Bias | High Risk of Bias | Uncertain Risk of Bias | Uncertain Risk of Bias | High Risk of Bias | Low Risk of Bias | Uncertain Risk of Bias |
| Currow, 1994 | High Risk of Bias | Uncertain Risk of Bias | Uncertain Risk of Bias | Uncertain Risk of Bias | High Risk of Bias | High Risk of Bias | Uncertain Risk of Bias |
| Dreher, 2007 | Uncertain Risk of Bias | Uncertain Risk of Bias | Uncertain Risk of Bias | Uncertain Risk of Bias | Low Risk of Bias | Low Risk of Bias | Uncertain Risk of Bias |
| Ducloux, 2007 | Low Risk of Bias | Uncertain Risk of Bias | Uncertain Risk of Bias | Uncertain Risk of Bias | High Risk of Bias | High Risk of Bias | Uncertain Risk of Bias |
| Duggleby, 2007 | Uncertain Risk of Bias | Uncertain Risk of Bias | Uncertain Risk of Bias | Uncertain Risk of Bias | Low Risk of Bias | Low Risk of Bias | High Risk of Bias |
| Dyar, 2012 | Uncertain Risk of Bias | Uncertain Risk of Bias | Uncertain Risk of Bias | Uncertain Risk of Bias | High Risk of Bias | Low Risk of Bias | Uncertain Risk of Bias |
| Edmonds, 2010 | Low Risk of Bias | Low Risk of Bias | Uncertain Risk of Bias | Uncertain Risk of Bias | Low Risk of Bias | High Risk of Bias | High Risk of Bias |
| Evangelista, 2012 | Uncertain Risk of Bias | Uncertain Risk of Bias | Uncertain Risk of Bias | Uncertain Risk of Bias | Low Risk of Bias | High Risk of Bias | Uncertain Risk of Bias |
| Farquhar, 2014 | Low Risk of Bias | Low Risk of Bias | High Risk of Bias | High Risk of Bias | Low Risk of Bias | Low Risk of Bias | Uncertain Risk of Bias |
| Flock, 2003 | Low Risk of Bias | Uncertain Risk of Bias | Low Risk of Bias | Low Risk of Bias | High Risk of Bias | Low Risk of Bias | Uncertain Risk of Bias |
| Galfin, 2012 | Low Risk of Bias | Uncertain Risk of Bias | Uncertain Risk of Bias | Uncertain Risk of Bias | Low Risk of Bias | High Risk of Bias | Uncertain Risk of Bias |
| Gammaitoni, 2000 | Low Risk of Bias | Uncertain Risk of Bias | Uncertain Risk of Bias | High Risk of Bias | Low Risk of Bias | High Risk of Bias | High Risk of Bias |
| Ganz, 1989 | Low Risk of Bias | Low Risk of Bias | Uncertain Risk of Bias | Uncertain Risk of Bias | Low Risk of Bias | Low Risk of Bias | Uncertain Risk of Bias |
| Giasson, 2012 | Uncertain Risk of Bias | Uncertain Risk of Bias | Uncertain Risk of Bias | Uncertain Risk of Bias | Low Risk of Bias | Low Risk of Bias | Uncertain Risk of Bias |
| Grande, 1999 | Low Risk of Bias | Low Risk of Bias | High Risk of Bias | High Risk of Bias | Low Risk of Bias | Low Risk of Bias | Uncertain Risk of Bias |
| Grimbert, 2004 | Uncertain Risk of Bias | Low Risk of Bias | Uncertain Risk of Bias | Uncertain Risk of Bias | Low Risk of Bias | Low Risk of Bias | Uncertain Risk of Bias |
| Gutgsell, 2013 | Low Risk of Bias | Low Risk of Bias | High Risk of Bias | Low Risk of Bias | Low Risk of Bias | Low Risk of Bias | Uncertain Risk of Bias |
| Hall, 2011 | Low Risk of Bias | Low Risk of Bias | High Risk of Bias | High Risk of Bias | High Risk of Bias | Low Risk of Bias | High Risk of Bias |
| Hall, 1998 | Low Risk of Bias | Low Risk of Bias | Uncertain Risk of Bias | Uncertain Risk of Bias | High Risk of Bias | Low Risk of Bias | Uncertain Risk of Bias |
| Hansen, 2014 | Low Risk of Bias | High Risk of Bias | Uncertain Risk of Bias | Uncertain Risk of Bias | Low Risk of Bias | High Risk of Bias | Uncertain Risk of Bias |
| Hardy, 2010 | Uncertain Risk of Bias | Low Risk of Bias | Low Risk of Bias | Low Risk of Bias | High Risk of Bias | Low Risk of Bias | Uncertain Risk of Bias |
| Hilliard, 2004 | Uncertain Risk of Bias | Uncertain Risk of Bias | High Risk of Bias | Uncertain Risk of Bias | Low Risk of Bias | High Risk of Bias | High Risk of Bias |
| Higginson, 2008 | Low Risk of Bias | Low Risk of Bias | High Risk of Bias | Low Risk of Bias | Low Risk of Bias | Low Risk of Bias | Uncertain Risk of Bias |
| Homs, 2005 | Uncertain Risk of Bias | Uncertain Risk of Bias | Uncertain Risk of Bias | Uncertain Risk of Bias | Low Risk of Bias | High Risk of Bias | High Risk of Bias |
| Hopkinson, 2013 | Low Risk of Bias | Uncertain Risk of Bias | Uncertain Risk of Bias | Uncertain Risk of Bias | High Risk of Bias | Low Risk of Bias | Uncertain Risk of Bias |
| Horne-Thompson, 2010 | Uncertain Risk of Bias | Low Risk of Bias | Low Risk of Bias | Low Risk of Bias | Low Risk of Bias | High Risk of Bias | Uncertain Risk of Bias |
| Hudson, 2000 | Low Risk of Bias | Low Risk of Bias | Uncertain Risk of Bias | Uncertain Risk of Bias | High Risk of Bias | High Risk of Bias | Uncertain Risk of Bias |
| Hudson, 2014 | Low Risk of Bias | Uncertain Risk of Bias | Uncertain Risk of Bias | Low Risk of Bias | Low Risk of Bias | Low Risk of Bias | Uncertain Risk of Bias |
| Israel, 2014 | Low Risk of Bias | Uncertain Risk of Bias | Uncertain Risk of Bias | Uncertain Risk of Bias | High Risk of Bias | Low Risk of Bias | Uncertain Risk of Bias |
| Jordhøy, 2006 | Low Risk of Bias | Uncertain Risk of Bias | Uncertain Risk of Bias | Uncertain Risk of Bias | Low Risk of Bias | High Risk of Bias | High Risk of Bias |
| Julião, 2009 | Low Risk of Bias | Low Risk of Bias | High Risk of Bias | Low Risk of Bias | High Risk of Bias | High Risk of Bias | High Risk of Bias |
| Kamboj, 2008 | Low Risk of Bias | Low Risk of Bias | Low Risk of Bias | Low Risk of Bias | Low Risk of Bias | High Risk of Bias | Uncertain Risk of Bias |
| Kissane, 2006 | Low Risk of Bias | Low Risk of Bias | High Risk of Bias | High Risk of Bias | Low Risk of Bias | Low Risk of Bias | Uncertain Risk of Bias |
| Kress, 1998 | Low Risk of Bias | Uncertain Risk of Bias | Uncertain Risk of Bias | Uncertain Risk of Bias | Low Risk of Bias | Low Risk of Bias | Low Risk of Bias |
| Kutner, 2000 | Low Risk of Bias | Low Risk of Bias | Low Risk of Bias | Low Risk of Bias | High Risk of Bias | Low Risk of Bias | Uncertain Risk of Bias |
| Kyle, 2012 | Low Risk of Bias | Low Risk of Bias | Uncertain Risk of Bias | Uncertain Risk of Bias | Low Risk of Bias | High Risk of Bias | High Risk of Bias |
| Latimer, 2012 | Uncertain Risk of Bias | Uncertain Risk of Bias | Uncertain Risk of Bias | Uncertain Risk of Bias | High Risk of Bias | High Risk of Bias | Uncertain Risk of Bias |
| Laval, 2012 | Low Risk of Bias | Uncertain Risk of Bias | Uncertain Risk of Bias | Uncertain Risk of Bias | Low Risk of Bias | Low Risk of Bias | High Risk of Bias |
| Laval, 2011 | Uncertain Risk of Bias | Uncertain Risk of Bias | Low Risk of Bias | Low Risk of Bias | Low Risk of Bias | High Risk of Bias | High Risk of Bias |
| LeCaer, 2004 | Uncertain Risk of Bias | Uncertain Risk of Bias | High Risk of Bias | High Risk of Bias | Low Risk of Bias | Low Risk of Bias | Uncertain Risk of Bias |
| Lee, 1999 | Low Risk of Bias | Low Risk of Bias | Low Risk of Bias | Low Risk of Bias | High Risk of Bias | High Risk of Bias | Uncertain Risk of Bias |
| Lim, 2007 | Low Risk of Bias | Uncertain Risk of Bias | Uncertain Risk of Bias | Uncertain Risk of Bias | Low Risk of Bias | Low Risk of Bias | Uncertain Risk of Bias |
| Lindholm, 2011 | Low Risk of Bias | Uncertain Risk of Bias | Uncertain Risk of Bias | Uncertain Risk of Bias | Uncertain Risk of Bias | Low Risk of Bias | Uncertain Risk of Bias |
| Link, 2010 | Uncertain Risk of Bias | Uncertain Risk of Bias | Uncertain Risk of Bias | Uncertain Risk of Bias | Uncertain Risk of Bias | High Risk of Bias | Uncertain Risk of Bias |
| Lundholm, 2005 | Low Risk of Bias | Uncertain Risk of Bias | Uncertain Risk of Bias | Uncertain Risk of Bias | Low Risk of Bias | High Risk of Bias | Uncertain Risk of Bias |
| Matlock, 2009 | Low Risk of Bias | Uncertain Risk of Bias | Uncertain Risk of Bias | Uncertain Risk of Bias | Low Risk of Bias | Low Risk of Bias | Uncertain Risk of Bias |
| Menahem, 2005 | Uncertain Risk of Bias | Uncertain Risk of Bias | Low Risk of Bias | Low Risk of Bias | High Risk of Bias | High Risk of Bias | Uncertain Risk of Bias |
| Miller, 2012 | Uncertain Risk of Bias | Uncertain Risk of Bias | High Risk of Bias | High Risk of Bias | Low Risk of Bias | High Risk of Bias | High Risk of Bias |
| Mills, 2013 | Low Risk of Bias | Uncertain Risk of Bias | Uncertain Risk of Bias | Uncertain Risk of Bias | High Risk of Bias | Low Risk of Bias | Uncertain Risk of Bias |
| Mystakidou, 2006 | Uncertain Risk of Bias | Uncertain Risk of Bias | Uncertain Risk of Bias | Uncertain Risk of Bias | Low Risk of Bias | High Risk of Bias | Uncertain Risk of Bias |
| Mok, 2010 | Low Risk of Bias | Low Risk of Bias | Uncertain Risk of Bias | Uncertain Risk of Bias | High Risk of Bias | Low Risk of Bias | Uncertain Risk of Bias |
| Nava, 2004 | Low Risk of Bias | Low Risk of Bias | High Risk of Bias | High Risk of Bias | Low Risk of Bias | Low Risk of Bias | Uncertain Risk of Bias |
| Philip, 2004 | Uncertain Risk of Bias | Uncertain Risk of Bias | Low Risk of Bias | Low Risk of Bias | Low Risk of Bias | Low Risk of Bias | High Risk of Bias |
| Popa-Velea, 1998 | Uncertain Risk of Bias | Uncertain Risk of Bias | Uncertain Risk of Bias | Uncertain Risk of Bias | Uncertain Risk of Bias | High Risk of Bias | High Risk of Bias |
| Prentice, 2000 | Low Risk of Bias | Uncertain Risk of Bias | Low Risk of Bias | Low Risk of Bias | Low Risk of Bias | High Risk of Bias | Uncertain Risk of Bias |
| Rabow, 2006 | High Risk of Bias | High Risk of Bias | Uncertain Risk of Bias | Uncertain Risk of Bias | Low Risk of Bias | High Risk of Bias | High Risk of Bias |
| Ramesh, 2014 | Uncertain Risk of Bias | Uncertain Risk of Bias | High Risk of Bias | High Risk of Bias | High Risk of Bias | High Risk of Bias | Uncertain Risk of Bias |
| Ranson, 2003 | Low Risk of Bias | Uncertain Risk of Bias | Uncertain Risk of Bias | Uncertain Risk of Bias | Low Risk of Bias | Low Risk of Bias | Uncertain Risk of Bias |
| Reck, 2012 | Low Risk of Bias | Uncertain Risk of Bias | Uncertain Risk of Bias | Uncertain Risk of Bias | Low Risk of Bias | High Risk of Bias | Uncertain Risk of Bias |
| Reinhardt, 2010 | Uncertain Risk of Bias | Uncertain Risk of Bias | Uncertain Risk of Bias | Low Risk of Bias | Low Risk of Bias | High Risk of Bias | Uncertain Risk of Bias |
| Reymond, 2003 | Uncertain Risk of Bias | Low Risk of Bias | Low Risk of Bias | Uncertain Risk of Bias | High Risk of Bias | High Risk of Bias | Uncertain Risk of Bias |
| Salas, 2015 | Low Risk of Bias | Uncertain Risk of Bias | Uncertain Risk of Bias | Uncertain Risk of Bias | Low Risk of Bias | Low Risk of Bias | Uncertain Risk of Bias |
| Sampson, 2009 | Low Risk of Bias | Low Risk of Bias | Uncertain Risk of Bias | Uncertain Risk of Bias | High Risk of Bias | Low Risk of Bias | Uncertain Risk of Bias |
| Schofield, 2004 | Uncertain Risk of Bias | Uncertain Risk of Bias | Uncertain Risk of Bias | Low Risk of Bias | Low Risk of Bias | Low Risk of Bias | High Risk of Bias |
| Sidebottom, 2008 | Uncertain Risk of Bias | Uncertain Risk of Bias | Uncertain Risk of Bias | Uncertain Risk of Bias | Low Risk of Bias | Low Risk of Bias | High Risk of Bias |
| Slatkin, 2013 | Low Risk of Bias | Uncertain Risk of Bias | Low Risk of Bias | Uncertain Risk of Bias | Low Risk of Bias | Low Risk of Bias | Uncertain Risk of Bias |
| Soden, 2010 | Uncertain Risk of Bias | Low Risk of Bias | Low Risk of Bias | Low Risk of Bias | Low Risk of Bias | High Risk of Bias | High Risk of Bias |
| Steinhauser, 2009 | Uncertain Risk of Bias | Uncertain Risk of Bias | Uncertain Risk of Bias | Low Risk of Bias | High Risk of Bias | High Risk of Bias | Uncertain Risk of Bias |
| Suh, 2002 | Low Risk of Bias | Low Risk of Bias | Low Risk of Bias | Low Risk of Bias | Low Risk of Bias | Low Risk of Bias | High Risk of Bias |
| Temel, 1994 | Low Risk of Bias | Uncertain Risk of Bias | High Risk of Bias | High Risk of Bias | Low Risk of Bias | Low Risk of Bias | Uncertain Risk of Bias |
| Thomas, 2012 | Uncertain Risk of Bias | Uncertain Risk of Bias | High Risk of Bias | High Risk of Bias | Low Risk of Bias | Low Risk of Bias | Uncertain Risk of Bias |
| Todd, 1998 | Low Risk of Bias | Uncertain Risk of Bias | High Risk of Bias | High Risk of Bias | Low Risk of Bias | Low Risk of Bias | Uncertain Risk of Bias |
| Toscani, 1990 | Uncertain Risk of Bias | Uncertain Risk of Bias | High Risk of Bias | Low Risk of Bias | Low Risk of Bias | High Risk of Bias | Uncertain Risk of Bias |
| Uitdehaag, 2013 | Low Risk of Bias | High Risk of Bias | Low Risk of Bias | Low Risk of Bias | High Risk of Bias | Low Risk of Bias | Uncertain Risk of Bias |
| Tsai, 2008 | Uncertain Risk of Bias | Uncertain Risk of Bias | Uncertain Risk of Bias | Uncertain Risk of Bias | Low Risk of Bias | Low Risk of Bias | High Risk of Bias |
| Tse, 2008 | Uncertain Risk of Bias | Uncertain Risk of Bias | Uncertain Risk of Bias | Uncertain Risk of Bias | Low Risk of Bias | High Risk of Bias | Uncertain Risk of Bias |
| Varela, 2004 | Low Risk of Bias | Uncertain Risk of Bias | High Risk of Bias | High Risk of Bias | High Risk of Bias | Low Risk of Bias | Uncertain Risk of Bias |
| Ventafridda, 2014 | Uncertain Risk of Bias | Uncertain Risk of Bias | High Risk of Bias | Low Risk of Bias | High Risk of Bias | High Risk of Bias | Uncertain Risk of Bias |
| Vogel, 2014 | Low Risk of Bias | Uncertain Risk of Bias | Uncertain Risk of Bias | Uncertain Risk of Bias | Low Risk of Bias | Low Risk of Bias | Uncertain Risk of Bias |
| Watanabe, 2000 | Low Risk of Bias | Low Risk of Bias | Low Risk of Bias | Low Risk of Bias | Low Risk of Bias | Low Risk of Bias | Low Risk of Bias |
| Weber, 1999 | Low Risk of Bias | Uncertain Risk of Bias | High Risk of Bias | High Risk of Bias | Low Risk of Bias | High Risk of Bias | High Risk of Bias |
| Wilcock, 2012 | Low Risk of Bias | Low Risk of Bias | Uncertain Risk of Bias | Low Risk of Bias | High Risk of Bias | High Risk of Bias | High Risk of Bias |
| Wu, 1998 | Uncertain Risk of Bias | Uncertain Risk of Bias | Uncertain Risk of Bias | Uncertain Risk of Bias | Uncertain Risk of Bias | High Risk of Bias | Uncertain Risk of Bias |
| Zimmermann, 2011 | Low Risk of Bias | Low Risk of Bias | Low Risk of Bias | High Risk of Bias | High Risk of Bias | Low Risk of Bias | High Risk of Bias |
