# Supplementary material for: Clinical trials in palliative care: a systematic review of their methodological characteristics and of the quality of their reporting
Source: BMC Palliat Care. 2017 Jan 25;16:10. doi: 10.1186/s12904-016-0181-9 (PMC5264484; doi:10.1186/s12904-016-0181-9)
Supplement: Additional file 4: — Criteria for judging “other risk of bias” in the Cochrane Risk of bias assessment tool. (DOCX 57 kb) [file 12904_2016_181_MOESM4_ESM.docx]

**Web appendix 4**

Criteria for judging “other risk of bias” in the Cochrane Risk of bias assessment tool

| Criteria for a judgement of ‘Low risk’ of bias | The study appears to be free of other sources of bias |
| --- | --- |
| Criteria for the judgement of ‘High risk’ of bias | There is at least one important risk of bias. For example, the study:   - Had a potential source of bias related to the specific study design used; or - Has been claimed to have been fraudulent; or - Had some other problem |
| Criteria for the judgement of  ‘Unclear risk’ of bias | There may be a risk of bias, but there is either:   - Insufficient information to assess whether an important risk of bias exists; or - Insufficient rationale or evidence that an identified problem will introduce bias |
